# Baseline serum uric acid level is associated with progression-free survival, disease control rate, and safety in postoperative patients with colorectal cancer treated by FOLFOX, FOLFIRI, or XELOX

**DOI:** 10.3389/fonc.2022.918088

**Published:** 2022-07-25

**Authors:** Xi Zhang, Qing-hong Chen, Ying Yang, Jing-xin Lin, Yan-chun Li, Tian-yu Zhong, Jie Chen, Si-qi Wu, Xiao-hu Chen, Rui-si Zhou, Jia-man Lin, Dong-qing Wang, Qiu-xing He, Yan-ting You, Xing-hong Zhou, Qiang Zuo, Yan-yan Liu, Jing-ru Cheng, Yi-fen Wu, Xiao-shan Zhao

**Affiliations:** ^1^Syndrome Laboratory of Integrated Chinese and Western Medicine, School of Chinese Medicine, Southern Medical University, Guangzhou, China; ^2^Department of Oncology, Affiliated Dongguan People’s Hospital, Southern Medical University, Dongguan, China; ^3^Department of Nephrology, the First Affiliated Hospital of Zhengzhou University, Zhengzhou, China; ^4^Department of Oncology, Nanfang Hospital, Southern Medical University, Guangzhou, China

**Keywords:** uric acid, colorectal cancer, prognosis, disease control rate, safety

## Abstract

**Background:**

High serum uric acid (SUA) levels increase the risk of overall cancer morbidity and mortality, particularly for digestive malignancies. Nevertheless, the correlation between SUA level and clinical outcomes of the postoperative patients with colorectal cancer (CRC) treated by chemotherapy is unclear. This study aimed at exploring the relationship between baseline SUA level and progression-free survival (PFS), disease control rate (DCR), and safety in postoperative CRC patients receiving chemotherapy.

**Patients and Methods:**

We conducted a retrospective study to evaluate the relationship between baseline SUA level and PFS, DCR, and incidence of serious adverse events of 736 postoperative CRC patients treated with FOLFOX, FOLFIRI or XELOX at our center.

**Results:**

Data from our center suggested that high baseline SUA level is linked to poor PFS in non-metastatic CRC patients using FOLFOX (HR=2.59, 95%CI: 1.29-11.31, p=0.018) and in male patients using FOLFIRI (HR=3.77, 95%CI: 1.57-39.49, p=0.012). In patients treated by FOLFIRI, a high SUA is also linked to a low DCR (p=0.035). In patients using FOLFOX, high baseline SUA level is also linked to a high incidence of neutropenia (p=0.0037). For patients using XELOX, there is no significant correlation between SUA level and PFS, effectiveness, or safety.

**Conclusions:**

These findings imply that a high SUA level is a promising biomarker associated with poor PFS, DCR and safety of postoperative CRC patients when treated with FOLFOX or FOLFIRI.

## Introduction

Colorectal cancer (CRC) is one of the most common cancers and ranks third in incidence and second in mortality among all the cancer types (1). Postoperative chemotherapy is critical for lowering the postoperative recurrence rate and extending the survival of CRC patients (2). Currently, the main systemic regimens used for postoperative chemotherapy in CRC are FOLFOX, FOLFIRI, and XELOX, but an increasing number of studies has reported the resistance to chemotherapeutic agents in postoperative CRC patients (3, 4). Uric acid (UA) belongs to the oxidative metabolites of purine nucleotides, and serum uric acid (SUA) is one of the most prevalent antioxidant molecules in human blood as a free radical scavenger and transition metal ion chelator (5, 6). SUA is primarily excreted from the body *via* the kidneys and gut (7). Elevated SUA has recognized pathogenic roles in respiratory and renal diseases (8, 9). In addition, clinical studies have shown that high SUA levels increase the risk of overall cancer morbidity and mortality, with digestive malignancies being particularly evident (10–13). UA is also reported to be one of the risk factors for the development of metabolic syndrome-associated colorectal adenomas (14). In patients with lung squamous cell carcinoma, esophageal cancer, gastric cancer, hepatitis B-associated liver cancer, breast cancer, acute myeloid leukemia, or diffuse large B-cell lymphoma, high SUA levels are associated with a poor prognosis (15–21). Furthermore, higher SUA levels prior to surgery are linked with a poorer prognosis in CRC patients (22). Elevated SUA levels are associated with metastasis in rectal cancer patients who have not received chemotherapy (23). SUA levels are significantly elevated in patients with metastatic CRC who responded well to bevacizumab chemotherapy, and this elevation is linked to better overall survival (24). SUA levels are not correlated with the prognosis of CRC patients receiving cetuximab chemotherapy (25). Elevated SUA before chemotherapy is associated with lower OS in patients with small cell lung cancer (26). Nonetheless, the impact of UA on the prognosis, efficacy and safety of CRC patients treated with chemotherapy after operation remains unclear. Thus, it’s urgent to elucidate the relationship between baseline SUA levels and their prognosis, disease control rate (DCR) and safety in patients treated with various chemotherapy regimens following CRC surgery.

## Methods

### Patients selection

We performed a retrospective study with the data of CRC postoperative patients treated with FOLFOX, FOLFIRI or XELOX in the Nanfang Hospital of Southern Medical University from November 2007 to April 2020. Medical practice data were collected by two independent investigators and assessed further by another investigator.

### Inclusion criteria

The inclusion criteria were as follows: (1) Patients who were diagnosed with CRC. (2) Patients received postoperative chemotherapy with FOLFOX, FOLFIRI or XELOX. (3) Patients had baseline SUA level after surgery.

### Exclusion criteria

The exclusion criteria were as follows: (1) Patients had events that significantly affected baseline SUA levels or were taking uric acid-lowering drugs. (2) Patients suffered from other malignant tumors. (3) Patients suffered from other diseases that seriously affect survival, such as uncontrolled hypertension, severe organ failure, other chronic diseases with long-term non-standard treatment, etc. (4) Patients with ECOG scores equal or above 3; (5) Patients detected baseline SUA 7 days or more later after the onset of chemotherapy. (6) Patients conducted imaging tests after the start of chemotherapy or 8 weeks before chemotherapy. (7) Patients with absence of baseline or endpoint imaging data.

### Basis for grouping

High SUA level was defined as >420 mmol/L in men and >357 mmol/L in women while the non-high SUA level was defined as ≤420 mmol/L in men and ≤357 mmol/L in women.

### Patients follow-up

The recruited patients treated with different chemotherapy regimens were followed-up. The primary end point of this study was progression-free survival (PFS), which was defined as the time from the first treatment with FOLFOX or FOLFIRI or XELOX following CRC surgery to the date of progression or death. If patients were still in a non-progressive state at the last follow-up, the end point of PFS was the date of the last follow-up. According to the Response Evaluation Criteria in Solid Tumors (RECIST) (version1.0), progression disease (PD) was defined as a 20% rise in the sum of the greatest diameter of the target lesions at baseline, or the occurrence of new lesions or confirmed advancement of non-target lesions (27).

### DCR

DCR, that refers to the proportion of all non-progressive patients at the end of follow-up in all patients included in the trial, was used to assess the efficacy of various chemotherapy regimens.

### Safety

We evaluated the safety of various chemotherapy regimens by the incidence of serious adverse events, which was defined as the proportion of patients with grade 3 or higher adverse events among all patients included in the study during the follow-up period. In this investigation, we recorded only adverse events with a grade of not less than three according to the Common Terminology Criteria for Adverse Events (CTCAE) version 5.0 (28).

### Statistical analysis

Categorical data were presented as number (%) and assessed by the Fisher’s exact test or the chi-square test when appropriate. Continuous data were presented as mean ± standard deviation (SD) and assessed by the student’s t-test with the GraphPad Prism (version 6.0). The log-rank test was used to compare survival curves of each group, and log-rank method was used to calculate hazard ratio (HR) and 95%CI.

## Results

### Baseline characteristics of included patients

A total of 736 patients treated with chemotherapy after CRC surgery were included in our clinical study after screening using inclusion and exclusion criteria, including 151 patients treated with FOLFOX, 45 patients treated with FOLFIRI, and 540 patients treated with FOLFOX. At baseline, there was no significant statistical difference between the HUA and non-HUA groups in terms of age, gender, primary tumor location, or tumor state (**Table 1**).

**Table:** Table 1 Baseline characteristics of colorectal cancer patients treated with postoperative chemotherapy.

Characteristic	FOLFOX			FOLFIRI			XELOX		
	Non-HUA(n=122)	HUA(n=29)	P value	Non-HUA(n=35)	HUA(n=10)	P value	Non-HUA(n=421)	HUA(n=119)	P value
Sex—no. (%)									
Male	79 (64.75)	19 (65.52)	0.94	19 (54.29)	6 (60.00)	1.00	277 (65.80)	86 (72.27)	0.18
Female	43 (34.43)	10 (34.48)		16 (45.71)	4 (40.00)		144 (34.20)	33 (27.73)	
Age—years	50.32 ± 1.11	51.76 ± 2.21	0.57	51.94 ± 1.75	47.90 ± 5.39	0.49	53.32 ± 0.58	55.35 ± 0.95	0.093
Site of primary tumor —no. (%)									
Right colon	29 (23.77)	4 (13.79)	0.24	8 (22.86)	1 (10.00)	0.56	78 (18.53)	26 (21.85)	0.43
Left colon or rectum	93 (76.23)	25 (86.21)		26 (74.29)	9 (90.00)		340 (80.76)	91 (76.47)	
Indistinguishable	–	–		1 (2.86)	0 (0)		3 (0.71)	2(1.68)	
Cancer state in baseline —no. (%)									
Non-mCRC	40 (32.79)	11 (37.93)	0.60	2 (5.71)	0 (0)	1.00	303 (71.97)	86 (72.27)	0.95
mCRC	82 (67.21)	18 (62.07)		33 (94.29)	10 (100.00)		118 (28.03)	33 (27.73)	

HUA, high uric acid; Non-HUA, non-high uric acid; mCRC, metastatic colorectal cancer; Non-mCRC, non- metastatic colorectal cancer.

### Prognosis of patients with FOLFOX

**Figure 1:**
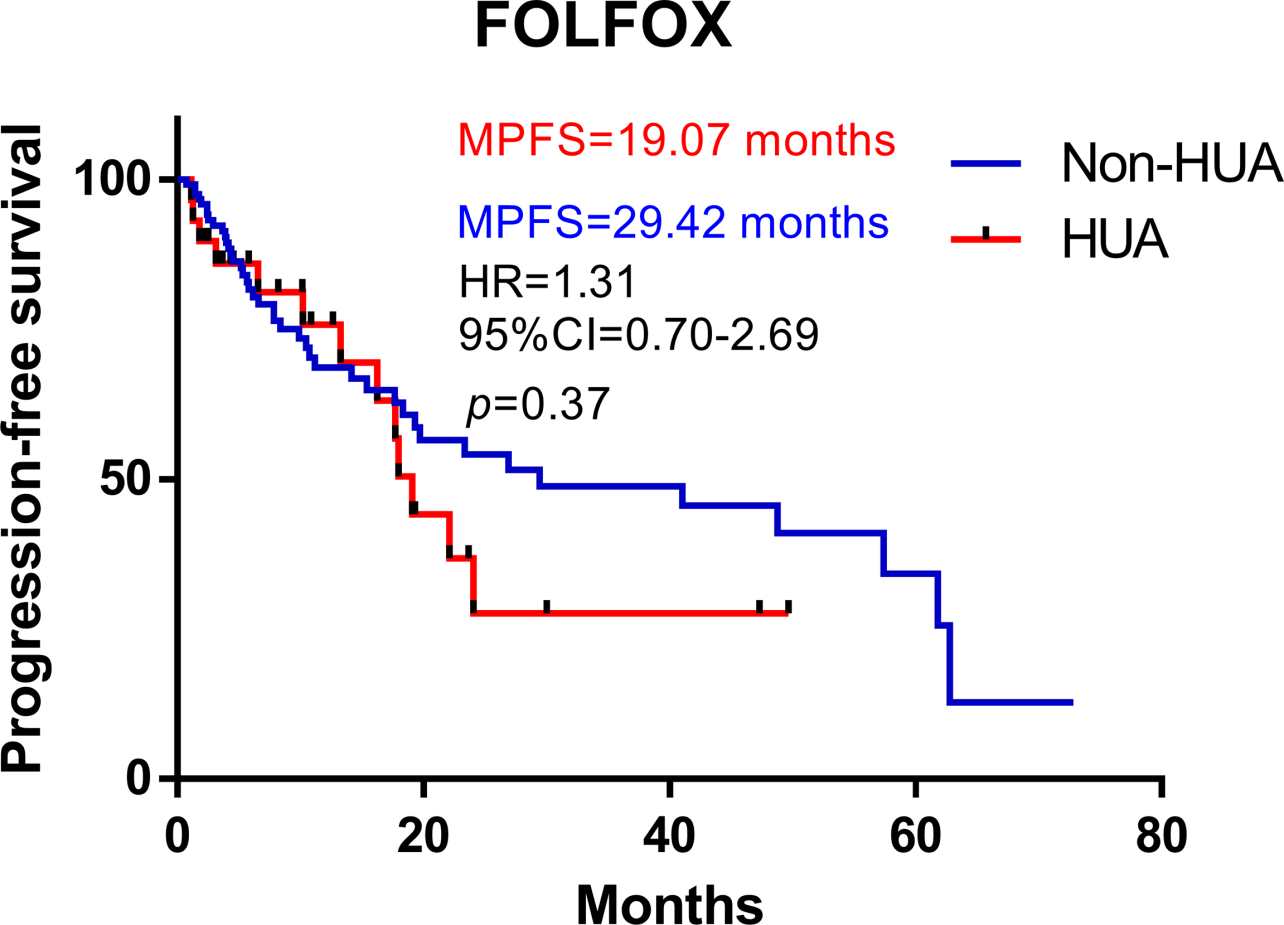
Kaplan-Meier plots comparing the PFS of the different baseline SUA level CRC patients treated with FOLFOX. HUA, high uric acid, Non-HUA, non-high uric acid, MPFS, median PFS.

**Figure 2:**
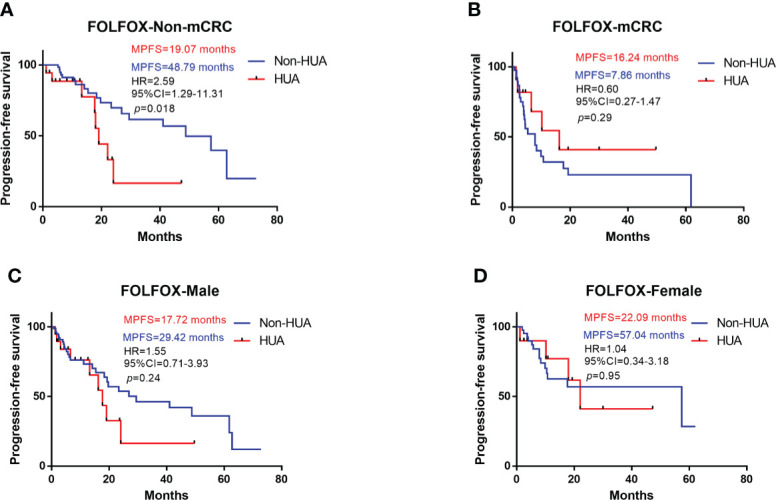
Subgroup analysis for PFS of patients treated with FOLFOX. HUA, high uric acid, Non-HUA, non-high uric acid, MPFS, median PFS. **(A)** PFS of the non-mCRC patients treated with FOLFOX. **(B)** PFS of the mCRC patients treated with FOLFOX. **(C)** PFS of male CRC patients treated with FOLFOX. **(D)** PFS of female CRC patients treated with FOLFOX.

In all patients treated with FOLFOX after operation, higher SUA levels were associated with shorter PFS (median PFS: 19.07 vs 29.42 months in the non-HUA group; HR: 1.31, 95%CI= (0.70-2.69), *p*=0.37, **Figure 1**). Nonetheless, this association was significant in non-metastatic CRC (non-mCRC) patients (median PFS: 19.07 vs 48.79 months in the non-HUA group; HR: 2.59, 95%CI= (1.29-11.31), *p*=0.018, **Figure 2A**) but not in metastatic CRC (mCRC) patients (median PFS: 16.24 vs 7.86 months in the non-HUA group; HR: 0.60, 95%CI= (0.27-1.47), *p*=0.29, **Figure 2B**). In the subgroup analysis of gender, higher SUA levels were associated with shorter PFS in both men(median PFS: 19.07 vs 29.42 months in the non-HUA group; HR: 1.55, 95%CI= (0.71-3.93), *p*=0.24, **Figure 2C**) and women (median PFS: 22.09 vs 57.40 months in the non-HUA group; HR: 1.04, 95%CI= (0.34-3.18), *p*=0.95, **Figure 2D**), although the association was not significant in both genders.

### Prognosis of patients with FOLFIRI

**Figure 3:**
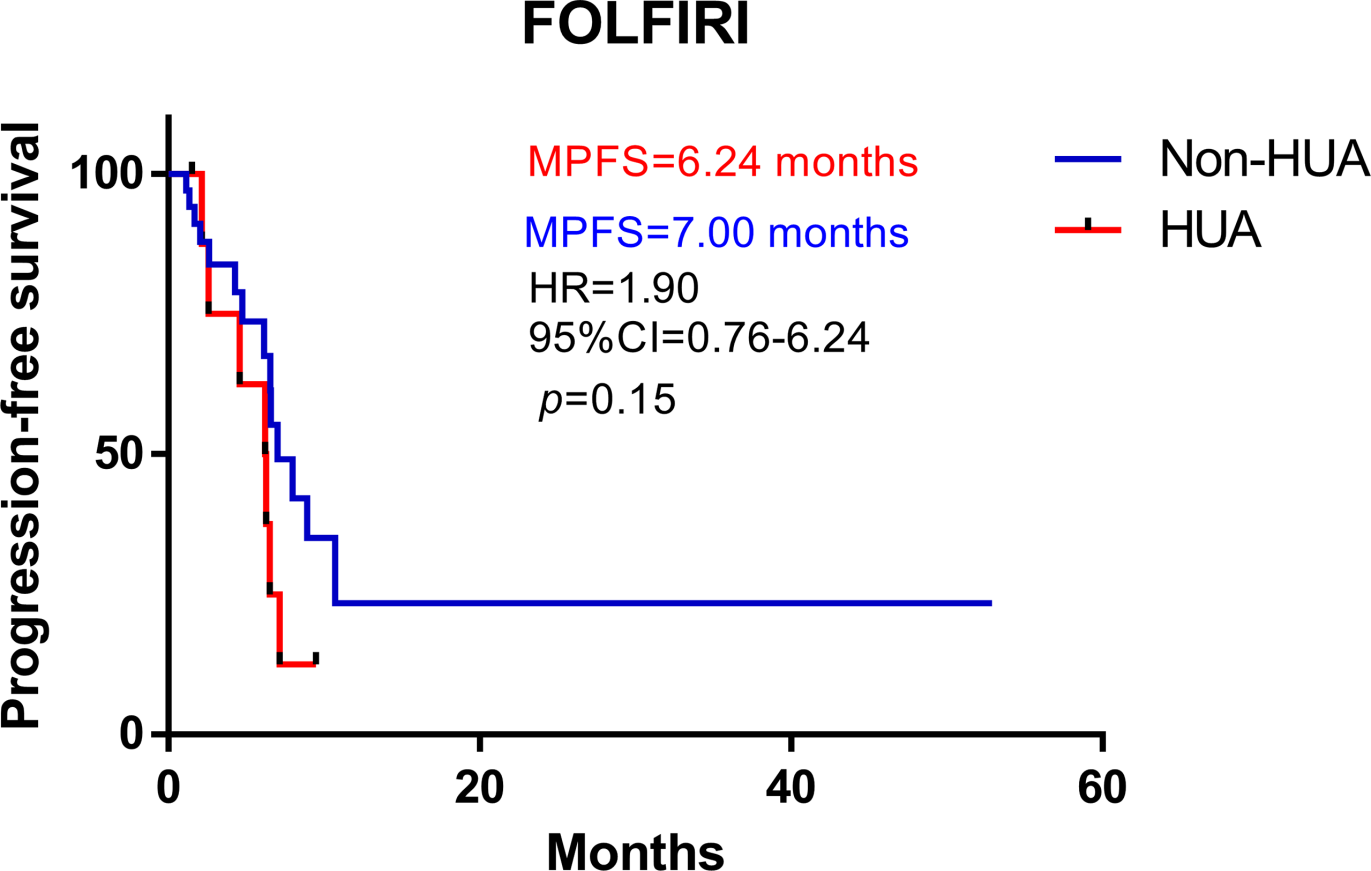
Kaplan-Meier plots comparing the PFS of the different baseline SUA level CRC patients treated with FOLFIRI. HUA, high uric acid, Non-HUA, non-high uric acid, MPFS, median PFS.

**Figure 4:**
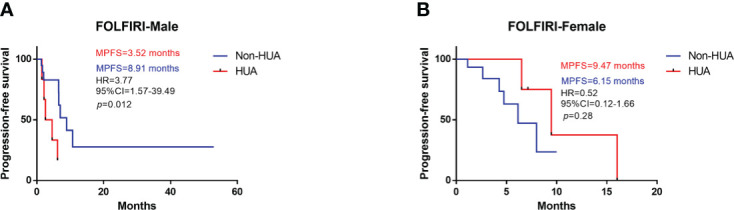
Subgroup analysis for PFS of patients treated with FOLFIRI. HUA, high uric acid, Non-HUA, non-high uric acid, MPFS, median PFS. **(A)** PFS of male CRC patients treated with FOLFIRI. **(B)** PFS of female CRC patients treated with FOLFRI.

In all patients treated with FOLFIRI following surgery, higher SUA levels were associated with shorter PFS (median PFS: 6.24 vs 7.00 months in the non-HUA group; HR: 1.90, 95%CI= (0.76-6.24), p=0.15, **Figure 3**). This association, however, was only significant in male patients (median PFS: 3.52 vs 8.91 months in the non-HUA group; HR: 3.77, 95%CI= (1.57-39.49), p=0.012, **Figure 4A**), but not in female patients (median PFS: 9.47 vs 6.15 months in the non-HUA group; HR: 0.52, 95%CI= (0.12-1.66), *p*=0.28, **Figure 4B**).

### Prognosis of patients with XELOX

**Figure 5:**
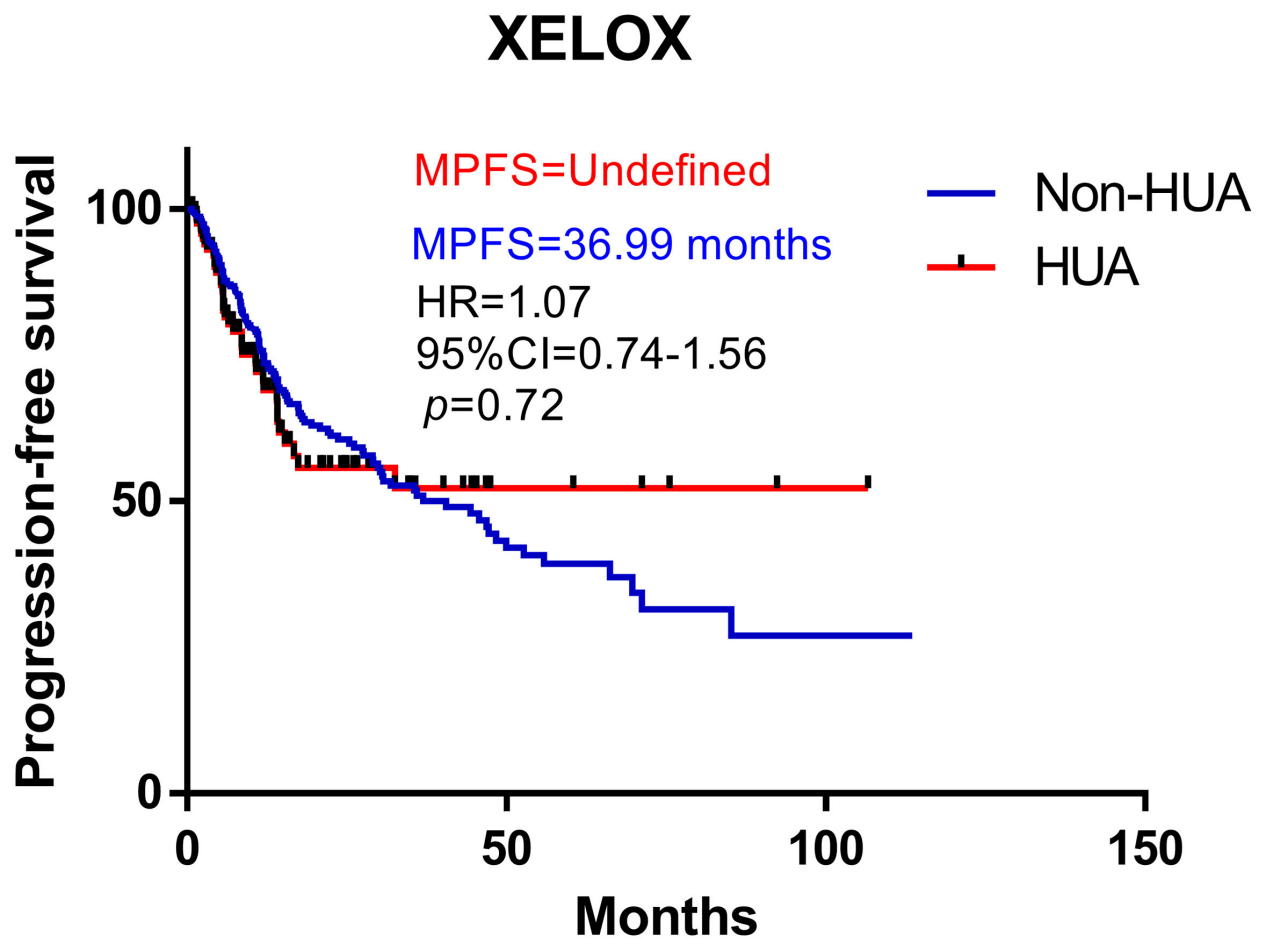
Kaplan-Meier plots comparing the PFS of the different baseline SUA level CRC patients treated with XELOX. HUA, high uric acid, Non-HUA, nonhigh uric acid, MPFS, median PFS.

**Figure 6:**
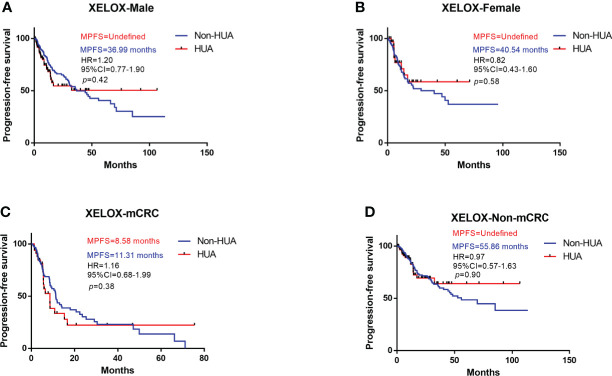
Subgroup analysis for PFS of patients treated with XELOX. HUA, high uric acid, Non-HUA, non-high uric acid, MPFS, median PFS. **(A)** PFS of male CRC patients treated with XELOX. **(B)** PFS of female CRC patients treated with XELOX. **(C)** PFS of the mCRC patients treated with XELOX. **(D)** PFS of the non-mCRC patients treated with XELOX.

Survival analysis suggests higher SUA levels were correlated with shorter PFS in all patients treated with XELOX chemotherapy after surgery (HR: 1.07, 95%CI= (0.74-1.56), *p*=0.72, **Figure 5**). Subgroup analysis revealed no significant difference in PFS between HUA group and non-HUA group in men, women, mCRC patients or non-metastatic CRC patients (**Figure 6**).

### Disease control rate of FOLFOX

Patients receiving FOLFOX chemotherapy after surgery had a larger DCR in the non-HUA group (65.57%) than in the HUA group (55.17%), but no significant difference in DCR was identified between the two groups (*p*=0.30, **Table 2**). In terms of DCR in males, women, mCRC patients, and non-metastatic CRC patients, subgroup analysis based on gender and cancer state revealed no significant difference between non-HUA and HUA groups in men, women, metastatic patients, or non-metastatic patients (*p*>0.05, **Table 2**).

**Table 2:** Relationship between SUA levels and disease control rate in patients using FOLFOX following colorectal cancer surgery.

	Non-HUA	HUA	P value
FOLFOX (n, %)			
DC	80 (65.57)	16 (55.17)	0.30
PD	42 (34.43)	13 (44.83)	
FOLFOX-male (n, %)			
DC	51 (64.56)	10 (52.63)	0.34
PD	28 (35.44)	9 (47.37)	
FOLFOX-female (n, %)			
DC	29 (67.44)	6 (60.00)	0.72
PD	14 (32.56)	4 (40.00)	
FOLFOX-mCRC (n, %)			
DC	16 (40.00)	6 (54.55)	0.50
PD	24 (60.00)	5 (45.45)	
FOLFOX-non-mCRC (n, %)			
DC	64 (78.05)	10 (55.56)	0.073
PD	18 (21.95)	8 (44.44)	

UA, high uric acid; Non-HUA, non-high uric acid; mCRC, metastatic colorectal cancer; non-mCRC, non- metastatic colorectal cancer; DC, disease control; PD, progression disease.

### Disease control rate of FOLFIRI

Patients who received FOLFIRI chemotherapy following surgery had a significantly greater DCR in the non-HUA group (60.00%) than the HUA group (20.00%) (*p*=0.035, **Table 3**). In terms of DCR in male and female patients, subgroup analysis based on gender indicated no significant difference between non-HUA and HUA groups (*p*>0.05, **Table 3**).

**Table 3:** Relationship between SUA levels and disease control rate in patients using FOLFIRI following colorectal cancer surgery.

	Non-HUA	HUA	P value
FOFIRI (n, %)			
DC	21 (60.00)	2 (20.00)	***0.035***
PD	14 (40.00)	8 (80.00)	
FOFIRI -male (n, %)			
DC	11 (57.59)	1 (16.67)	0.16
PD	8 (42.11)	5 (83.33)	
FOFIRI -female (n, %)			
DC	10 (62.50)	1 (25.00)	0.28
PD	6 (37.50)	3 (75.00)	

HUA, high uric acid; Non-HUA, non-high uric acid; DC, disease control; PD, progression disease. The italic and bold values indicate p-values < 0.05.

### Disease control rate of XELOX

There was no significant difference in DCR between the non-HUA group and the HUA group of patients receiving XELOX after surgery (*p*>0.05, **Table 4**). Subgroup analysis based on gender and cancer state suggests no significant difference between non-HUA group and HUA group in terms of DCR in men, women, metastatic patients, or non-metastatic patients (*p*>0.05, **Table 4**).

**Table 4:** Relationship between SUA levels and disease control rate in patients using XELOX following colorectal cancer surgery.

	Non-HUA	HUA	P value
XELOX (n, %)			
DC	284 (67.46)	83 (69.75)	0.47
PD	137 (32.54)	36 (30.25)	
XELOX-male (n, %)			
DC	192 (69.31)	59 (68.60)	0.90
PD	85 (30.69)	27 (31.40)	
XELOX-female (n, %)			
DC	92 (63.89)	24 (72.73)	0.34
PD	52 (36.11)	9 (27.27)	
XELOX-mCRC (n, %)			
DC	56 (47.46)	14 (42.42)	0.61
PD	62 (52.54)	19 (57.58)	
XELOX-Non-mCRC (n, %)			
DC	228 (75.24)	69 (80.23)	0.34
PD	75 (24.75)	17 (19.77)	

UA, high uric acid; Non-HUA, non-high uric acid; mCRC, metastatic colorectal cancer; non-mCRC, non- metastatic colorectal cancer; DC, disease control; PD, progression disease.

### Safety of patients with FOLFOX

In patients treated with FOLFOX following surgery, grade 3 or higher adverse events were recorded, including liver failure, neutropenia, intestinal obstruction, and thrombocytopenia. The HUA group had a greater incidence of neutropenia than the non-HUA group (p=0.0037, **Table 5**). In addition, the incidence of liver failure and intestinal obstruction were higher in the HUA group than in the non-HUA group and the incidence of thrombocytopenia was lower than in the non-HUA group, but there was no significant difference between the two groups in the incidence of adverse events mentioned above (p>0.05, **Table 5**).

**Table 5:** Relationship between baseline SUA levels and safety in colorectal cancer patients treated with postoperative chemotherapy.

Adverse events(grade ≥3)	FOLFOX			FOLFIRI				XELOX		
	Non HUA (n=122)	HUA (n=29)	P value	Non-HUA (n=35)		HUA (n=10)	P value	Non-HUA (n=421)	HUA (n=119)	P value
Liver failure	1 (0.82)	1 (3.45)	0.35	–		–	–	12 (2.85)	0 (0)	0.078
Neutropenia	4 (3.28)	6 (20.69)	***0.0037***	5 (14.29)		1 (10.00)	1.00	4 (0.95)	2 (1.68)	0.62
Intestinal obstruction	1 (0.82)	2 (6.90)	0.095	–		–	–	3 (0.71)	0 (0)	1.00
Thrombocytopenia	2 (1.64)	0 (0)	1.00	–		–	–	–	–	–
Vomiting	–	–	–	1 (2.86)		0 (0)	1.00	–	–	–
Anorexia	–	–	–	1 (2.86)		0 (0)	1.00	–	–	–
Anemia	–	–	–	–		–	–	1 (0.24)	0 (0)	1.00
Myelosuppression	–	–	–	–		–	–	2 (0.48)	0 (0)	1.00
Diarrhea	–	–	–	–		–	–	0 (0)	1 (0.84)	0.22

HUA, high uric acid; Non-HUA, non-high uric acid. The italic and bold values indicate p-values < 0.05.

### Safety of patients with FOLFIRI

Grade three or higher adverse events, including neutropenia, vomiting, and anorexia were observed in patients receiving FOLFIRI chemotherapy after surgery. But there was no significant difference in the occurrence of these adverse events between HUA patients and non-HUA individuals (p>0.05, **Table 5**).

### Safety of patients with XELOX

In patients treated with XELOX chemotherapy after surgery, we observed grade 3 or higher adverse events, including neutropenia, intestinal obstruction, anemia, myelosuppression, and diarrhea. In the HUA group, the incidence of neutropenia and diarrhea was higher than in the non-HUA group, while the incidence of liver failure, intestinal obstruction, anemia, and bone marrow suppression was lower than in the non-HUA group, but there was no significant difference in the incidence of these adverse events between the two groups (p>0.05, **Table 5**).

## Discussion

This is the first study to show a linkage between the baseline SUA levels and prognosis, effectiveness, and safety in patients treated with chemotherapy following CRC resection. Other study shows that patients with higher SUA levels were found to have a higher percentage and shorter duration of brain metastases, as well as a lower overall survival rate in non-small cell lung cancer patients (15). While CRC patients in stage IIIA/IIIB who have a high SUA level may have early metastasis independent of all variables, implying uric acid is associated with CRC metastasis (29). In line with these findings, we found that non-metastasis CRC patients who were treated with FOLFOX following CRC surgery had shorter PFS if they had high baseline SUA than those had low baseline SUA. FOLFIRI is primarily used for mCRC patients who are refractory to oxaliplatin chemotherapy, and it has been shown to improve quality of life and prolong survival in these patients (30–32). As a result, 43 patients with mCRC and only 2 patients with non-metastatic CRC were enrolled in FOLFIRI chemotherapy after surgery. Our data show that the DCR of patients treated with FOLFIRI after CRC was considerably greater in those with high baseline SUA levels than in those with low baseline SUA levels. This may be related to the fact that uric acid promotes the CRC progression or reduces the drug sensitivity to 5-FU. In addition, we found that the PFS of men with high SUA levels on this regimen were significantly shorter than those with low SUA levels; while in women, there was no statistically significant difference in PFS with different SUA levels. We hypothesize that the above discrepancies in different genders are due to the effect of estrogen on the uric acid levels. Estrogen may promote uric acid excretion by regulating uric acid transport-related proteins (33, 34). By suppressing xanthine oxidase and maintaining a stable nutrient metabolism, estrogen can lower uric acid generation (35, 36). Therefore, for female patients with higher SUA levels, estrogen may reduce UA to a level below the threshold that promotes CRC progression or reduces the sensitivity of CRC to 5-FU, leading to a non-significant difference in DCR and prognosis.

When SUA level is elevated, both in the state of soluble high uric acid and crystalline uric acid, ROS production and IL-1β formation can be increased, resulting in oxidative stress and inflammatory response and thus promoting tumor progression (37–39). Increased ROS is associated to an increase in matrix metalloproteinase (MMP) in the tumor microenvironment, which is directly related to tumor invasion and migration (40, 41). In addition, elevated ROS accelerates angiogenesis by inducing the production of angiogenic factors (42).Therefore, ROS production and IL-1β may be responsible for shortening PFS in patients with high SUA levels. As a capecitabine-based regimen, XELOX differs from the regularly used 5-FU-based chemotherapy regimen of FOLFOX and FOLFIRI. As an oral fluorouracil agent, capecitabine is slightly different from 5-FU that directly targets tumor cells and it has low bioavailability. Capecitabine enhances the concentration of 5-Fu in tumor tissues by utilizing the different activities of thymine phosphorylase in tumor tissues and normal tissues (43). Studies have shown that the average concentration of 5-FU in primary colorectal tumors is 3.2 times higher than that in adjacent normal tissues, and the mean ratio of 5-FU concentration in liver metastasis to normal tissues is 1.4. The average 5-FU concentration ratio of colorectal tumor tissue to plasma is over 20, while that of other tissues are between 8 and 10 (44). Based on our data, there was no significant difference in prognosis or DCR between patients with varying SUA levels who received XELOX following surgery. This discrepancy could be due to the fact that the concentration of 5-Fu in CRC tissues in XELOX patients was higher than in FOLFOX or FOLFIRI patients, masking the reduction in CRC sensitivity to 5-FU induced by elevated uric acid.

In our study, patients receiving FOLFOX chemotherapy for CRC experienced grade 3 or higher adverse effects, such as liver failure, neutropenia, intestinal obstruction, and thrombocytopenia. These events are common in people treated by FOLFOX (45–47). Neutropenia is a regular occurrence in patients during postoperative chemotherapy, which is also one of the serious adverse events of chemotherapy. Our data show that patients with high SUA levels had considerably higher incidences of neutropenia than those with low SUA levels. High concentrations of SUA increase intracellular oxidation (6). In addition, by activating calpain-1 and endoplasmic reticulum stress (ERS), excessive UA induces apoptosis in normal cells (48). As a result, we hypothesize that the increased prevalence of neutropenia in patients with high SUA levels could be linked to the fact that UA enhances the apoptotic action of chemotherapeutic medicines on neutrophils. There was no significant association between the occurrence of adverse events and blood UA levels in individuals who received FOLFIRI as postoperative chemotherapy. We speculate that this is due to the sample size being insufficient. For patients treated with XELOX, we have not observed significant association between the incidence of adverse events and SUA levels either, which could be due to the difference in the mechanism of action of capecitabine and 5-FU.

Our research has some limitations. We only focused on the values of baseline SUA, and failed to include the SUA levels after chemotherapy. Secondly, the current study was based on retrospective observations, future prospective studies with larger samples are needed to confirm our findings. Thirdly, the number of high SUA patients was much less than that of non-HUA patients, and this may lead to the inaccuracy of the statistics analysis. Further studies with larger sample size or multicenter studies are needed to validate our findings.

In summary, our findings imply that the baseline blood uric acid level is an important biomarker correlated with the clinical prognosis, DCR, and safety of postoperative chemotherapy for CRC. Elevated baseline SUA is associated with poor prognosis in non-metastatic CRC patients treated by FOLFOX and in male patients treated by FOLFIRI, low DCR in patients with FOLFIRI, and reduced safety in patients with FOLFOX.

## Data availability statement

The original contributions presented in the study are included in the article/supplementary material. Further inquiries can be directed to the corresponding authors.

## Ethics statement

The studies involving human participants were reviewed and approved by the Chinese Ethics Committee of Registering Clinical Trials. Written informed consent for participation was not required for this study in accordance with the national legislation and the institutional requirements.

## Author contributions

XZ, Q-hC, YY, J-xL, and Y-cL: conceptualization, data curation, formal analysis, investigation, software, and visualization. XZ: methodology and writing–original draft. T-yZ, JC, S-qW, X-hC, R-sZ, J-mL, D-qW, Q-xH, Y-tY, X-hZ, QZ, Y-yL, and J-rC: writing–review and editing. X-sZ and Y-fW: funding acquisition, project administration, resources, and supervision. All authors contributed to the article and approved the submitted version.

## Funding

This work was supported by the Key Project of National Natural Science Foundation of China (81830117), the National Natural Science Foundation of China (81803877, 81873205), the Natural Science Foundation of Guangdong Province, China (2020B1515120063, 2021A1515110990), and the Innovation Team and Talents Cultivation Program of National Administration of Traditional Chinese Medicine (ZYYCXTD-C-202001).

## Acknowledgments

We are very grateful to Professor Hiu Yee Kwan from Hong Kong Baptist University for polishing our manuscript.

## Conflict of interest

The authors declare that the research was conducted in the absence of any commercial or financial relationships that could be construed as a potential conflict of interest.

## Publisher’s note

All claims expressed in this article are solely those of the authors and do not necessarily represent those of their affiliated organizations, or those of the publisher, the editors and the reviewers. Any product that may be evaluated in this article, or claim that may be made by its manufacturer, is not guaranteed or endorsed by the publisher.
